# Hypoxic Microenvironment-Induced Reduction in PTEN-L Secretion Promotes Non-Small Cell Lung Cancer Metastasis through PI3K/AKT Pathway

**DOI:** 10.1155/2022/6683104

**Published:** 2022-03-02

**Authors:** Xuyang Song, Jinxi He, Bingqing Shi, Yuning Han

**Affiliations:** General Thoracic Surgery, General Hospital of Ningxia Medical University, Yinchuan 750001, Ningxia Province, China

## Abstract

**Objective:**

Lung cancer is the leading cause of cancer-related deaths worldwide. The aim of this study was to investigate the effects of hypoxic microenvironment on PTEN-L secretion and the effects of PTEN-L on the metastasis of non-small cell lung cancer (NSCLC) and the potential mechanisms.

**Methods:**

The expression levels of PTEN-L in NSCLC tissues, cells, and cell culture media were detected. The transfection of PTEN-L overexpression construct or HIF-1*α*-siRNAs was conducted to manipulate the expression of PTEN-L or HIF-1*α*. NSCLC cells were introduced into 200 *μ*M CoCl2 medium for 72 hours under 37°C to simulate hypoxia. The proliferation and apoptosis of the A549 cells were determined by the Cell Counting Kit-8 assay and Annexin V-FITC/PI-stained flow cytometry assay, respectively. Wound healing assay and transwell invasion assay were used to measure the migration and invasion of A549 cells. The protein expression of PTEN, PTEN-L, PI3K/AKT pathway-related proteins, and HIF-1*α* was detected by Western blot.

**Results:**

PTEN and PTEN-L are downregulated in lung cancer tissues and cells. The protein expression of PTEN-L in the culture medium of lung cancer cell lines is decreased. The hypoxic microenvironment inhibits PTEN-L secretion. The low level of PTEN-L promotes cell proliferation, migration, and invasion, as well as inhibits apoptosis of A549 cells. The overexpression of PTEN-L attenuated the activation of the PI3K/AKT pathway by the hypoxic microenvironment. The knockdown of HIF-1*α* upregulates PTEN-L secretion under hypoxia.

**Conclusions:**

The hypoxic microenvironment inhibits PTEN-L secretion and thus activates PI3K/AKT pathway to induce proliferation, migration, and invasion promotion, and apoptosis inhibition in NSCLC cells.

## 1. Introduction

Lung cancer is one of the most dreaded malignant tumors and the leading cause of cancer-related deaths across the world (approximately a quarter of cancer deaths) [1, 2]. Non-small cell lung cancer (NSCLC) is the most commonly reported subtypes of lung cancer, which represents 85% of all lung cancer [3]. The strong invasive and metastatic nature of NSCLC leads to poor prognosis [4]. Despite developments in conventional (chemo)radiotherapy and surgery, the survival of NSCLC patients remains poor [5]. Thus, it is an increasingly urgent need to illuminate the molecular mechanisms underlying and new effective therapeutic strategies of NSCLC.

The TME represents a dynamic cellular milieu in which the tumor exists [6, 7]. TME composition differs by cancer types, yet signature characteristics comprise immune cells, stromal cells, blood vessels, and extracellular matrix. Hypoxia is present in most tumors and is caused by an imbalance between high oxygen consumption and insufficient oxygen delivery capacity [8]. Hypoxia can trigger cell death through apoptosis/necrosis; however, what it can also do is protect against cell death via stimulating adaptive responses, which in reverse promotes cell proliferation or angiogenesis, thereby promoting tumor development [9]. Transcription factor hypoxia-inducible factor-1*α* (HIF-1*α*) has an essential effect on cancer cellular metabolism. HIF-1*α* promotes glycolysis and facilitates tumor progression [10]. Nevertheless, the underlying molecular mechanism involved in the effect of tumor growth and metastasis remains unclear.

PTEN (phosphatase and tensin homolog deleted on chromosome 10), a prominent tumor suppressor gene [11, 12], inhibits PI3K/AKT pathway via lipid phosphatase activity [13]. PTEN has a translation variant named PTEN-Long (PTEN-L) [14, 15]. PTEN-L consists of all the domains of PTEN with the added 173 N-terminal amino acids in its N-terminal alternatively translated region (ATR) [14]. In many diseases, hypoxia affects PTEN secretion and thus regulates the progression of diseases. In nonalcoholic fatty liver disease, hypoxia induces HIF-1*α* accumulation, and consequently, PTEN expression is reduced, exacerbating liver fibrosis in nonalcoholic fatty liver disease [16]. In neonatal hypoxic-ischemic brain damage, HIF-1*α* inhibition of PTEN mediates the protective function of BMSCs on neurons under hypoxia [17]. In brain ischemia, reciprocally opposed binding partners of PTEN-L or PTEN in cytosolic or nuclear components are regulated following ischemic-like stress induced by oxygen-glucose deprivation [18]. PTEN has an important effect on various tumors. Sementino et al. [19] suggested that reduced Tp53 and PTEN activity in mouse mesothelium cells promotes mesothelioma progression. Shi et al. [20] exhibited that miR-29a reduces proliferation and drug resistance of colon cancer cells through upregulation of PTEN. Yu et al. [21] discovered that PTEN promoted NSCLC metastasis via the integrin *α*V*β*6 pathway. Purified PTEN-L is taken up by tumor cells and regulates the PI3K/AKT pathway [22]. In vitro experiments also show that secreted PTEN-L inhibits the proliferation of U87 cells [23]. Based on these reports, we speculate that hypoxia may affect the metastasis of NSCLC via regulating the secretion of PTEN-L. The effect of PTEN-L on NSCLC cells remains unclear.

In our current research, we explored the role of hypoxic environments played in PTEN-L secretion and whether it has an impact on the progression of NSCLC. Understanding the effects of PTEN-L as an exogenous therapeutic agent in NSCLC contributes to the improvement of NSCLC.

## 2. Materials and Methods

### 2.1. Clinical Samples

The NSCLC tissues and paired normal paracancerous tissues were derived from tissues derived from 30 NSCLC patients undergoing surgical resection. Then, the samples were stored in a refrigerator at −80°C in time for subsequent experiments. All participants in this research were not receiving treatment prior to surgery. This study has been reviewed and approved by the Ethics Committee of the General Hospital of Ningxia Medical University Hospital.

### 2.2. Cell Culture and Treatment

The human bronchial epithelial cell line (16HBE) and the human NSCLC cell lines A549, A549, H226, and H460 were maintained in RPMI 1640 medium. The human NSCLC cell line SPC-A1 was cultivated in Dulbecco's modified Eagle medium (Gibco, USA). All media consisted of 10% fetal bovine serum (FBS) and antibiotics. All cell cultures were preserved in a humidified atmosphere at 37°C with 5% CO_2_. All these cell lines were obtained from the American Type Culture Collection (ATCC, Manassas, VA, USA). The protein expression levels of PTEN and PTEN-L in each cell and the level of PTEN-L secreted in the culture medium were detected.

To preliminarily analyze the effect of PTEN-L on NSCLC cells under the hypoxic microenvironment, A549 cells were divided into 4 groups: control group, hypoxia group, PTEN-L group, and hypoxia + PTEN-L group. The biological behavior of cells in each group was detected, and the changes in PI3K/AKT pathway in cells of each group were compared.

To analyze the effect of HIF-1*α* on the expression of PTEN-L under hypoxia, a rescue experiment was performed. A549 cells were divided into 4 groups: control, hypoxia, si-HIF-1*α*, and hypoxia + si-HIF-1*α*. The expression and secretion levels of PTEN-L in each group of cells were detected.

The control group cells were cultured under the above conditions. In vitro hypoxic microenvironment was induced by CoCl2. PTEN-L was overexpressed, and HIF-1*α* was silenced by transfection. The specific experimental methods were as follows.

### 2.3. Hypoxic Microenvironment

80% confluent NSCLC cells were cultured in 200 *μ*M CoCl2 medium (72 hours, 37°C) to simulate hypoxia. Following the treatment of CoCl2, we separated PTEN-L from the supernatant [24, 25].

### 2.4. Plasmid Constructs

The complementary DNAs (cDNAs) of PTEN-L were shown to be synthesized by reverse transcription and amplified using polymerase chain reaction (PCR). Then, the cDNA has been incorporated into pCMV-Tag2B vector (N-terminal Flag tag). The constructs of Flag-PTEN-L were generated by subcloning. All DNA sequencing was used to confirm plasmid constructs. The expression of plasmid was confirmed by Western blot.

### 2.5. Cell Transfection

One day prior to transfection, A549 cells were spread in a 24-well plate at a cell density of 1 × 10^5^ cells/well. Cells were transfected with plasmid or siRNA when they reached 80% confluence. We performed the transfection using Lipofectamine 3000 reagent (Invitrogen) according to the instructions, and the transfected cells were incubated in a cell culture incubator at 37°C for 48 hours.

### 2.6. Cell Counting Kit-8 (CCK-8) Assay

A549 cells with a density of 4 × 10^3^ cells/well were spread in a 96-well culture plate. 10 *μ*L CCK-8 (ImmunoWay Biotechnology Company) was added per 100 *μ*L medium and then cultured for 4 h at 37°C. The absorbance at 450 nm was measured by a microplate reader.

### 2.7. Annexin V-FITC/PI-Stained Flow Cytometry Assay

A549 cells were centrifuged (2000 rpm, 5 min) and resuspended (400 *μ*L binding buffer). Then, 5 ul of each Annexin V-FITC and PI was placed into the cell suspension. The cells and reagents are mixed and then left to stand (on ice, 10 min). Kaluza Analysis Software (Beckman Coulter, Inc.) was adopted to process the data of flow cytometry. The second quadrant and the fourth quadrant represent apoptotic cells.

### 2.8. Wound Healing Assay

We dispersed A549 cells into a 6-well plate (1 × 10^5^/well). A wound was generated by scratching the cell monolayer using a 200 *μ*l pipette gun tip when the A549 cells reached near 100% confluence. Cell fragments were taken off by PBS. The plates were imaged, followed by the addition of the serum-free medium, and the plates were imaged again by incubation at 37°C for 24 hours. The wound healing processes were observed under a light microscope (magnification, ×100; Olympus Corporation) at 0 and 48 h after the scratch, and the distance was evaluated with ImageJ software. The experiment was conducted independently in triplicate.

### 2.9. Transwell Assay

Cells were spread into the upper chamber of transwell plates with A549 (1 × 10^5^ cells per well) and cultured with serum-free medium. Serum-free medium containing cells was added to the basement membrane matrix-coated upper chamber. The lower chamber was filled with media supplemented containing 10% FBS. The transwell chambers were kept in the incubator for 24 h, and cotton swabs were employed for wiping the cells remaining on the membrane upper surface. The cells on the transwell membranes were fixed with paraformaldehyde (4%) for 10 min. The crystal violet solution (0.5%) was used to stain. A microscope with a magnification of 100 (Olympus) was taken to count the number of invading cells.

### 2.10. Western Blot

Protein was extracted from A549 cells using lysis buffer according to the lysis buffer protocol. The BCA Protein Concentration Assay Kit has been optimized for determining total protein content in protein samples. SDS-PAGE was used to isolate the proteins, and then, we performed gel electrophoresis experiments to transfer proteins to PVDF membranes. 1% BSA was applied to block the PVDF membrane (30 min, 25°C), which was subsequently incubated with the corresponding primary antibody, including anti-PTEN antibody (ab267787, 1 : 1000, Abcam, USA), anti-PI3K antibody (ab178860, 1 : 1000, Abcam, USA), anti-p-PI3K antibody (ab188570, 1 : 5000, Abcam, USA), anti-AKT antibody (ab3778; 1 : 200, Abcam, USA), anti-p-AKT antibody (ab52915, 1 : 10000, Abcam, USA), anti-HIF-1*α* antibody (ab179483, 1 : 1000, Abcam, USA), and anti-GAPDH antibody (ab32360, 1 : 5000, Abcam, USA) overnight at 4°C. GAPDH was used as internal controls. The horseradish peroxidase-labeled secondary antibody (ab6721, 1 : 2000, Abcam, USA) was adopted to conjugate to the primary antibody on the membrane (1 h, 25°C). The enhanced chemiluminescence (ECL) plus reagents (Beyotime) were used to display protein bands.

### 2.11. Statistical Analysis

The data were analyzed by SPSS software 23.0. Descriptive statistics were presented as the means ± SD. The method of comparison of quantitative variables between two groups was an unpaired *t*-test. Comparisons of qualitative variables between multiple groups were done by the one-way ANOVA. *P* < 0.05 was considered to be a significant difference.

## 3. Results

### 3.1. A Decrease in PTEN and PTEN-L Expression Was Observed in NSCLC Tissues and Cells

**Figure 1:**
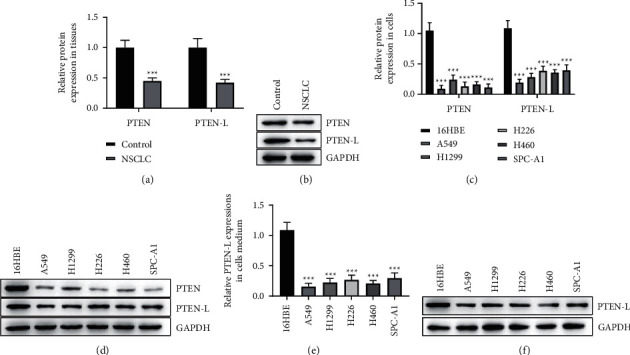
A decrease in PTEN and PTEN-L expression was observed in NSCLC tissues and cells. A, B, The relative protein expression of PTEN and PTEN-L in NSCLC tissues and para-carcinoma tissues (control) was examined by Western blot. C, D, The relative protein expression of PTEN and PTEN-L in normal lung epithelial cells (16HBE) and lung cancer cell lines (16HBE, A549, H226, H460, SPC-A1) was examined by Western blot. E, F, The relative protein expression of PTEN-L in the culture medium of normal lung epithelial cells (16HBE) and lung cancer cell lines (16HBE, A549, H226, H460, SPC-A1) was examined by Western blot. (^∗∗∗^*p* < 0.001 vs. control group or 16HBE group).

First, we examined the PTEN and PTEN-L levels in NSCLC tissues and cells and found a significantly lower level of PTEN and PTEN-L protein in the NSCLC tissues than that in paracancerous tissues (Figures 1(a), 1(b), *p* < 0.001). In vitro analysis of protein expression in cell lines showed that PTEN and PTEN-L protein was reduced in NSCLC cell lines (A549, H1299, H226, H460, SPC-A1) compared with the normal lung epithelial cells (16HBE) (Figure 1(c), 1(d), *p* < 0.001). Similarly, we also observed an apparent reduction in PTEN-L protein expression in the medium of NSCLC cell lines when compared to that of the lung epithelial cells, as presented in Figure 1(e), 1(f). The results suggested that PTEN and PTEN-L protein expression was decreased in NSCLC tissues and cells. The secreted PTEN-L might realize cell-cell communication and regulation.

### 3.2. Hypoxic Microenvironment Inhibits PTEN-L Secretion to Promote Proliferation and Inhibit Apoptosis of A549 Cells

**Figure 2:**
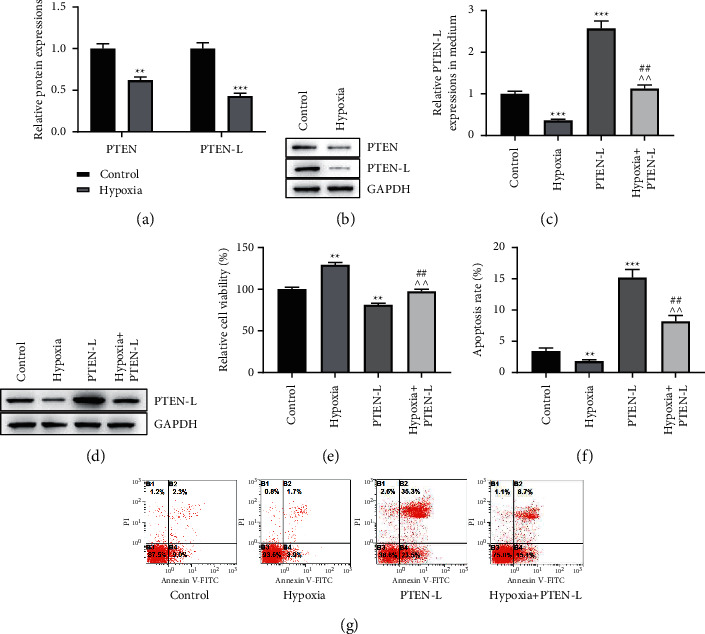
Hypoxic microenvironment inhibits PTEN-L secretion to promote proliferation and inhibit apoptosis of A549 cells. A, B, The effect of hypoxia on PTEN and PTEN-L protein expression was detected by Western blot. C, D, The relative protein expression of PTEN-L in A549 cell cultures under hypoxic and/or overexpressing PTEN-L was examined by Western blot. E The relative proliferation viability of A549 cells under hypoxic and/or overexpressing PTEN-L was examined by CCK-8 assay. F, G, Apoptosis of A549 cells under hypoxic and/or overexpressing PTEN-L was examined by double staining with Annexin V-FITC/propidium iodide (PI) and flow cytometry. (^∗∗^*p* < 0.01,^∗∗∗^*p* < 0.001 vs. control group; ^∧∧^*p* < 0.01 vs. hypoxia group; ^##^*p* < 0.01 vs. PTEN-L group).

Subsequently, we detected the effect of hypoxia on PTEN-L secretion and explored whether the excretive PTEN-L participates in the regulation of NSCLC cell proliferation and apoptosis. The findings demonstrated that the protein expression of PTEN and PTEN-L under hypoxic conditions was lower than that under normoxic conditions in A549 cells, suggesting that hypoxia could affect the secretion of PTEN and PTEN-L (Figures 2(a), 2(b), *p* < 0.01, *p* < 0.001). As shown in Figures 2(c), 2(d), hypoxia induced a reduction in PTEN-L protein level, while the overexpression of PTEN-L increased PTEN-L protein level in A549 cell medium. Hypoxia-mediated repression of PTEN-L protein level could be restored by the overexpression of PTEN-L (*p* < 0.01, *p* < 0.001). Besides, we also found that hypoxia induced the elevation of cell viability, whereas the upregulation of PTEN-L showed a decreased trend in cell proliferation. The overexpression of PTEN-L attenuated the hypoxia-induced increase in cell viability (Figure 2(e), *p* < 0.01). The apoptosis rate of A549 cells is decreased under hypoxic conditions, which could be strongly enhanced by upregulating PTEN-L. The upregulation of PTEN-L could partially reduce the increase in apoptosis rate caused by hypoxia (Figure 2(f), 2(g), *p* < 0.01, *p* < 0.001). The above experiments demonstrated that the hypoxic microenvironment restrained PTEN-L secretion to induce proliferation promotion and apoptosis inhibition in A549 cells. This suggested that NSCLC cells under hypoxic environment might regulate the malignant biological behavior of other cells through PTEN-L.

### 3.3. Hypoxic Microenvironment Inhibits PTEN-L Secretion to Promote Metastasis of NSCLC and Activate PI3K/AKT Signaling

**Figure 3:**
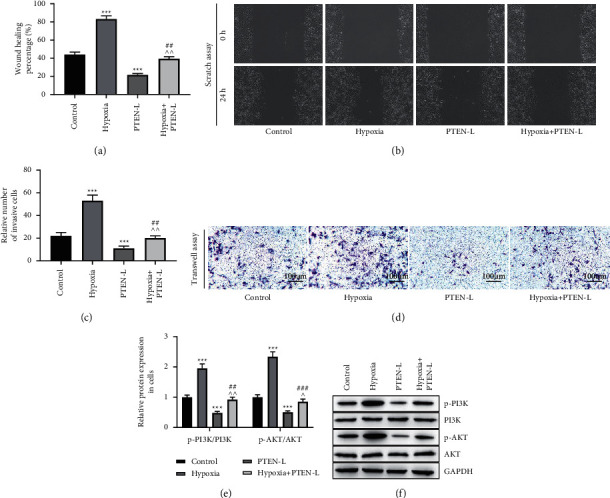
Hypoxic microenvironment inhibits PTEN-L secretion to promote migration and invasion of A549 cells and activate PI3K/AKT signaling. A, B, Wound healing assay to determine migration of A549 cells. C, D, Transwell invasion assay was performed to determine the invasion of A549 cells. E, F, Expression of PI3K/AKT pathway-related proteins was detected by Western blot. (^∗∗∗^*p* < 0.001 vs. control group; ^∧^*p* < 0.05,^∧∧^*p* < 0.01 vs. hypoxia group; ^##^*p* < 0.01,^###^*p* < 0.001 vs. PTEN-L group).

Furthermore, we validated the effect of the decreased PTEN-L secretion caused by hypoxic microenvironment on the migration and invasion of A549 cells. The wound healing assay indicated that the wound healing percentage of A549 cells was elevated in the hypoxic group and decreased in the PTEN-L group in contrast to the control group. The hypoxia-mediated increase in wound healing rate in A549 cells can be inhibited by the upregulation of PTEN-L (Figure 3(a), 3(b), *p* < 0.01, *p* < 0.001). The transwell assay results indicated that the relative number of invasion cells was increased under hypoxia and decreased by the overexpression of PTEN-L. The overexpression of PTEN-L can inhibit the increase caused by the overexpression of PTEN-L under normoxia (Figure 3(c), 3(d), *p* < 0.01, *p* < 0.001), which revealed that hypoxia-induced reduction in PTEN-L secretion promotes migration and invasion of A549 cells. Moreover, we detected the impact of hypoxic and PTEN-L on PI3K/AKT signaling. In contrast to the control group, the protein levels of p-PI3K/PI3K and p-AKT/AKT were upregulated in the hypoxic group and downregulated in the PTEN-L group; in the hypoxic + PTEN-L group, the upregulation of protein levels of p-PI3K/PI3K and p-AKT/AKT can be partially restored by the overexpression of PTEN-L (Figures 3(e), 3(f), *p* < 0.05, *p* < 0.01, *p* < 0.001), which demonstrated that the PI3K/AKT signaling may be participated in the regulation of the reduced PTEN-L secretion caused by hypoxic microenvironment, as well as the PTEN-L-mediated biological behavior of NSCLC cell.

### 3.4. Silencing HIF-1*α* Partially Reverses the Decrease in PTEN-L Secretion Induced by Hypoxic Environment

**Figure 4:**
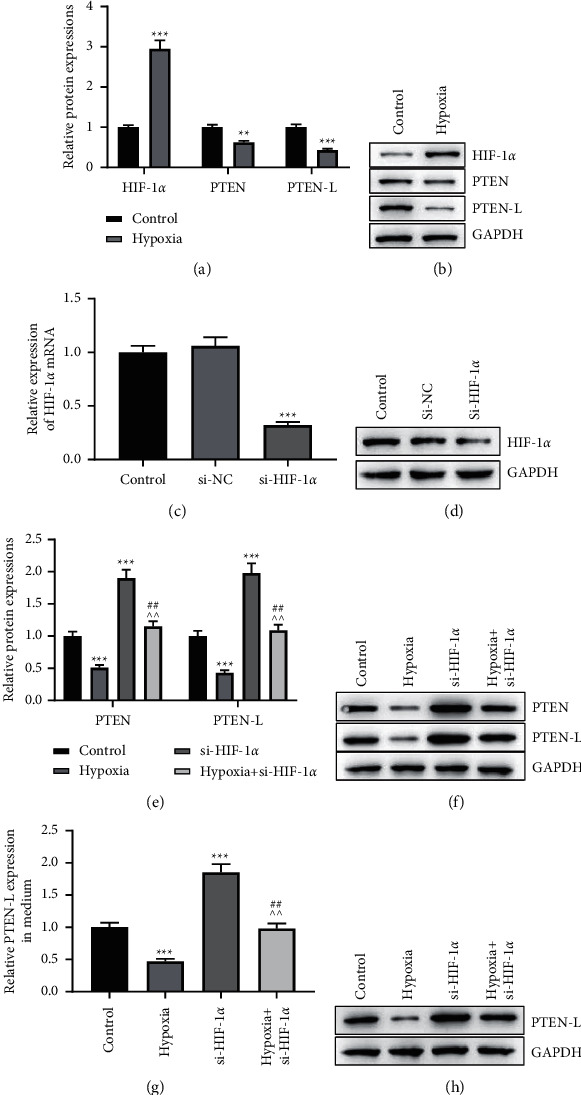
Silencing HIF-1*α* significantly reverses the decrease in PTEN-L and PTEN secretion-induced hypoxic environment. A, B, Relative protein expression of HIF-1*α*, PTEN, and PTEN-L was detected by Western blot in A549 cells. C, D, Transfection effect of si-HIF-1*α* was detected by Western blot in A549 cells. E, F, Relative protein expression of PTEN and PTEN-L was detected by Western blot in A549 cells. G, H, Relative protein expression of PTEN-L was detected by Western blot in the medium of A549 cells. (^∗∗^*p* < 0.01,^∗∗∗^*p* < 0.001 vs. control group; ^∧∧^*p* < 0.01 vs. hypoxia group; ^##^*p* < 0.01,^###^*p* < 0.001 vs. si-HIF-1*α* group).

Finally, we determined the effect of HIF-1*α* on PTEN-L and PTEN secretion under hypoxic environments in A549 cells. We found that HIF-1*α* expression was upregulated, whereas PTEN-L and PTEN-L expression was downregulated in hypoxic environments (Figures 4(a), 4(b), *p* < 0.01, *p* < 0.001). To determine whether HIF-1*α* regulates PTEN-L secretion under hypoxia, we knocked down HIF-1*α* (Figures 4(c), 4(d), *p* < 0.001). The results showed that the knockdown of HIF-1*α* can partially downregulate the elevation of PTEN and PTEN-L caused by hypoxia (Figures 3(e), 3(f), *p* < 0.01, *p* < 0.001). Similarly, when compared to the hypoxia group, the knockdown of HIF-1*α* also significantly reversed the decrease in PTEN-L protein in the medium of the A549 cells (Figures 3(g), 3(h), *p* < 0.01, *p* < 0.001). The above findings revealed that the knockdown of HIF-1*α* promotes PTEN-L secretion under hypoxia. This suggested that HIF-1*α* was a key factor regulating the expression of PTEN-L under the hypoxic microenvironment.

## 4. Discussion

Sanctuary of the devil tumor cells constantly interacts with the surrounding microenvironment [6]. The tumor microenvironment affects cancer characteristics, such as its ability to promote proliferation and angiogenesis and inhibit apoptosis and the immune system [26]. The glycolysis in cervical cancer can be facilitated by CNPY2 under anoxic conditions [27]. The expression of mmu_circ_0000826 was raised under hypoxia, which in turn facilitates the formation and metastatic ability of colorectal cancer [28]. Hypoxia is a key feature of solid tumors. Hypoxia induces the downregulation of FAM13A and thus reduces the proliferation and metastasis of NSCLC [29].

PTEN has been reported to be carcinogenic, and a number of biological processes such as cell proliferation, growth, migration, metabolism, and death are regulated by PTEN [30]. Many studies have demonstrated that PTEN can be secreted in extracellular vesicles of exosomes [31]. It can also be secreted as a naked protein (longer heterodimer) [31]. The second form secreted is called PTEN-Long [31]. Similar to PTEN, PTEN-Long can inhibit the PI3K-AKT pathway and restrains cellular proliferation [32].

One investigation found that PTEN expression was reduced under continuous hypoxic stimulation [33]. Combined treatment of pancreatic cancer with GAS1 and PTEN inhibited cell invasion promoting cell death [34]. The reduced expression of Tp53 and PTEN promotes the progression of pleural and peritoneal malignant mesotheliomas [19]. miR-29a targets P-gp downstream of PTEN to induce drug resistance, proliferation inhibition, and apoptosis promotion in colon cancer cells [20]. In this study, we found that PTEN and PTEN-L were downregulated in NSCLC tissues and cells and PTEN-L was downregulated in the medium of NSCLC cells. The finding that PTEN-L level is increased in paraneoplastic tissue cells backs up the results in our study [22]. The above findings indicate that PTEN and PTEN-L are likely to be involved in the development of NSCLC under hypoxic conditions.

To verify whether PTEN and PTEN-L under hypoxia are involved in the development of NSCLC, we examined cell proliferation, apoptosis, migration, and invasion of A549 cells. We found that the hypoxic microenvironment inhibits PTEN-L secretion to induce proliferation, migration, and invasion promotion, and apoptosis inhibition in NSCLC cells. The study found that lncRNA ZEB2-AS1 promotes the proliferation, migration, and invasion of NSCLC cells through downregulating PTEN [35]. PTEN inactivation contributes to metastasis of NSCLC [36]. PTEN facilitates metastasis of NSCLC via the integrin *α*V*β*6 pathway [21].

The intracellular signal transduction pathway PI3K/AKT is frequently overactivated in human cancers [37]. The PI3K/AKT pathway is related to the development and progression of a range of cancers [38–40]. Zhang et al. [41] discovered that PI3K/AKT is involved in cisplatin resistance in NSCLC. Zhang et al. [42] showed that PI3K/Akt signaling pathway inhibition enhanced AZD9291-induced cell death. Wei et al. [43] reported that LPCAT1 may promote NSCLC via PI3K/AKT pathway. In gastric cancer, the PI3K/AKT pathway is activated under hypoxic conditions, which further promotes epithelial-mesenchymal transition [44]. In human dental pulp cells, hypoxia activates the PI3K/AKT pathway and thus inhibits oxidative stress [44]. In lung cancer cells, hypoxia-induced activation of PI3K/AKT and ERK pathways antagonizes apoptosis [45]. PTEN utilizes its lipid phosphatase activity and dephosphorylation of PIP3 to inhibit tumor [46, 47]. We demonstrated that the overexpression of PTEN-L inhibited hypoxia-induced activation of PI3K/AKT signaling pathway. This suggests that hypoxia-mediated reduction in PTEN-L secretion is likely to mediate receptor tumor cell proliferation, metastasis, and apoptosis through activation of the PI3K/AKT pathway. A number of researches have reported the involvement of hypoxia-activated PI3K/AKT pathway in cancer progression. In colorectal cancer, hypoxia increases Nur77 expression to further activate PI3K/AKT pathway to induce EMT [48]. In glioma, the expression of PLOD2 is increased under hypoxia, which can promote tumor proliferation and metastasis through PI3K/AKT signaling [49]. In hepatocellular carcinoma, hypoxia promotes TUFT1 expression, which activates the Ca^2+^/PI3K/AKT pathway to promote cancer growth and metastasis [50].

Hypoxia plays a driving role in the development of tumors [49]. It has been reported by many studies that hypoxia-inducible factor-1*α* (HIF-1*α*) induces hypoxia and regulates tumor cell adaptation to hypoxia in response to changes in oxygen [51]. Stability and levels of HIF-1*α* in cancer cells are elevated in hypoxic environments [52]. The study found that hypoxia/HIF-1*α* suppresses antagonistic tumor immune responses and promotes malignant tumor development [53]. Therefore, we speculate that it is likely that HIF-1*α* is participated in the secretion of PTEN-L in the hypoxic microenvironment. Our results show that silencing HIF-1*α* can reverse the decrease in PTEN-L expression caused by hypoxia, suggesting that decreasing the level of HIF-1*α* promotes the secretion of PTEN-L under hypoxia. This suggests that cells in the hypoxic center of tumor tissue may regulate tumor cell proliferation and migration in normoxic environment by expressing PTEN-L. However, only cell experiments were performed in this study. The clinical value of PTEN-L in NSCLC deserves further analysis. The effect of secreted PTEN-L on cell-to-cell communication in NSCLC needs to be confirmed by further cell and animal experiments.

## 5. Conclusion

Hypoxia induces a decrease in PTEN-L secretion in NSCLC. Hypoxia-induced reduction in PTEN-L induces proliferation and metastasis promotion, and apoptosis inhibition in NSCLC. The PI3K/AKT pathway may be involved in the regulation of PTEN-L. Our study offers new insights into the role of PTEN-L on NSCLC development in response to hypoxic stimulation, which shows that PTEN-L is a new potential target for the treatment of NSCLC.

## Data Availability

The datasets used or analyzed during this study are available from the corresponding author on reasonable request.
